# Use of Denosumab in Children With Osteoclast Bone Dysplasias: Report of Three Cases

**DOI:** 10.1002/jbm4.10210

**Published:** 2019-08-22

**Authors:** Alexander Upfill‐Brown, Susan Bukata, Nicholas M Bernthal, Alan L Felsenfeld, Scott D Nelson, Arun Singh, Katherine Wesseling‐Perry, Fritz C Eilber, Noah C Federman

**Affiliations:** ^1^ Department of Orthopaedic Surgery University of California Los Angeles Los Angeles CA USA; ^2^ Department of Oral & Maxillofacial Surgery University of California Los Angeles Los Angeles CA USA; ^3^ Department of Pathology University of California Los Angeles Los Angeles CA USA; ^4^ Division of Hematology & Oncology, Department of Medicine University of California Los Angeles Los Angeles CA USA; ^5^ Division of Nephrology, Department of Pediatrics University of California Los Angeles Los Angeles CA USA; ^6^ Division of Surgical Oncology, Department of Surgery University of California Los Angeles Los Angeles CA USA

**Keywords:** Osteoclasts, Denosumab, RANKL, Hypercalcemia, Oncology

## Abstract

Denosumab has been used successfully to treat disease‐associated osteoclast overactivity, including giant cell tumor of bone. Given its mechanism of action, denosumab is a potent potential treatment of other osteoclast bone dysplasias including central giant cell granuloma (CGCG), aneurysmal bone cyst (ABC), and cherubism. Relatively little is known about the safety and efficacy of denosumab in patients with these conditions, especially in children. We report on 3 pediatric patients treated with denosumab over a 3‐year period at UCLA Medical Center (Los Angeles and Santa Monica, CA, USA): a 12‐year‐old with recurrent ABC of the pelvis, a 14‐year‐old with CGCG of the mandible, and a 12‐year‐old with cherubism. All were started on a 1‐year course of 15 doses 120 mg s.c., given monthly with two loading doses on day 8 and 15. All patients demonstrated rapid and pronounced clinical improvement while on denosumab, including a significant reduction in pain and sclerosis of lytic lesions on radiographs. Within 1 month of initiating therapy, 2 patients experienced hypocalcemia (Common Terminology Criteria for Adverse Events [CTCAE] grade 2) and hypophosphatemia, with 1 patient experiencing symptoms. One patient went on to experience symptomatic rebound hypercalcemia (CTCAE grade 4) 5 months after completing therapy, requiring bisphosphonates and calcitonin. For the second patient, we developed a schedule to wean denosumab involving the progressive lengthening of time between doses from 1 to 4 months in 1‐month increments before cessation. We found that denosumab therapy results in significant clinical and radiographic improvement for pediatric patients with nonresectable ABC, CGCG, and cherubism. Problems with serum calcium may be more common in younger patients, with symptomatic and protracted rebound hypercalcemia after cessation of therapy the most significant. We present a potential solution to this problem with progressive spacing of doses. Potential serious adverse events from alterations in calcium homeostasis should be explored in prospective clinical trials. © 2019 The Authors. *JBMR Plus* published by Wiley Periodicals, Inc. on behalf of American Society for Bone and Mineral Research.

## Introduction

Denosumab, a monoclonal antibody against RANKL, has been used successfully to treat diseases associated with osteoclast overactivity, including giant cell tumor of bone (GCTB), osteoporosis, and lytic lesions associated with bony metastases.^1^, ^2^, ^3^ Increasingly, denosumab has been used off‐label for other disorders of bone thought to result, at least in part, from similar osteoclastic pathology. These include central giant cell granuloma (CGCG), aneurysmal bone cyst (ABC), cherubism, and fibrous dysplasia (FD).

Cherubism is characterized by focal resorption and replacement with proliferative fibro‐osseous masses of the maxillary and mandibular bones. A gain‐of‐function mutation in SH3BP2 has been identified as a driver of cherubism; this mutation leads to osteoclastogenesis in the presence of RANKL and TNF‐α through interactions with a variety of proteins in hemopoietic progenitor cells.^4^ Similarly, osteoclast multinucleated giant cells have been identified in CGCG.^5^ These same cells have been found in pathological specimens of ABCs, though it is hypothesized they are reactive in nature.^6^, ^7^ Finally, excess osteoclastogenesis has been consistently identified in patients with FD.^8^ The potential role of multinucleated giant cells in these conditions has led to the limited application of denosumab in their treatment.

No clinical trials of denosumab have been published, and there are no ongoing clinical trials listed on Clinicaltrials.gov for these groups of patients. Cases have been reported for the use of denosumab in ABC,^7^, ^9^, ^10^, ^11^, ^12^, ^13^ CGCG,^14^, ^15^, ^16^, ^17^, ^18^ cherubism,^19^ and FD^20^, ^21^ for 8, 10, 1, and 3 patients, respectively. Of these, 5 patients were <16 years old when they began treatment. Although this therapy appears to be equally effective in younger patients based on this small sample, problems with calcium regulation may be more prevalent.

Though the safety of denosumab in adults and skeletally mature populations has been studied in patients with GCTB, little information exists on possible side‐effects in skeletally immature patients or patients with other pathology. These known side‐effects in adult populations include hypocalcemia (5%), hypophosphatemia (3%), back and or extremity pain (1%), and osteonecrosis of the jaw (1%).^22^ In pediatric patients, there have been two reports of hypercalcemia. In one 9‐year‐old patient with CGCG of the mandible, hypercalcemia was asymptomatic and resolved without intervention.^17^ In another 9‐year‐old patient with FD of the femur, severe symptomatic hypercalcemia developed after cessation of treatment requiring i.v. calcitonin and bisphosphonate therapy, returning to normal serum calcium levels after approximately 5 months.^21^

The use of denosumab in pediatric patients remains poorly studied; consequently clinicians are understandably reluctant to use this therapy in this population. Here we present our experience treating 3 pediatric patients off‐label with denosumab for a diagnosis of CGCG, ABC, or cherubism. We report a similar case of rebound hypercalcemia as reported previously,^21^ as well as our strategy for preventing this in our most recently treated patient.

## Patients and Methods

All patients were treated at UCLA Medical Centers in Los Angeles and Santa Monica, California, USA. Diagnosis was established using tissue histopathology, radiographic appearance, and clinical history. All patients weighed over 40 kg when therapy was initiated, and all were started on an adult dose of 120 mg s.c. every 4 weeks, with additional loading doses on day 8 and 15 in accordance with the dosing schedule established for adult patients.^2^, ^3^ The first patient, with recurrent ABC of the pelvis, started treatment when she was 12 years old, receiving 14 doses. The second patient, with CGCG of mandible, began therapy when he was 14 years old and received 15 doses. The third patient, with cherubism, began therapy when she was 12 years old and has received 13 doses to date. No patient had clinical or radiological signs of a wider syndrome.

All patients received daily calcium carbonate and vitamin D supplementation, initially with 500 mg calcium carbonate and 1000 units of vitamin D3 daily and increased as necessary. No restrictions were placed on diet or activity for patients with these pathologies. For the patients with extremity tumors, full weight‐bearing is allowed, but moderate‐to‐high impact exercise and sports is discouraged. Patient follow‐up schedules, laboratory studies, and imaging studies were not standardized and were established and administered at the discretion of the providers managing treatment. Plain film X‐rays were the standard imaging modality; CT and MRI were utilized where appropriate.

## Results

### Patient 1: recurrent aneurysmal bone cyst of the pelvis

This patient was a 10‐year‐old female who was diagnosed with an extensive ABC of the pelvis. She underwent curettage and bone grafting; she tolerated the procedure well. However, 2 years later she presented with pain following a fall; it was found that her pelvic ABC had recurred, hence she underwent a second curettage and bone grafting. Histology revealed a cystic bone lesion with connective tissue, reactive woven bone, and hemorrhage, with fibroblasts and scattered osteoclast‐type giant cells noted. Her case was reviewed by a multidisciplinary musculoskeletal tumor board: The consensus was that an additional ABC was present, and that the lesion was not amenable to repeat curettage without significant potential morbidity. At a weight of 40 kg, she initiated denosumab therapy 1 month prior to her 13th birthday. She received denosumab on a monthly schedule with a day 8 loading dose (patient missed a day 15 loading dose) of 120 mg over the course of a year.

She achieved excellent clinical response and showed sclerosis of lesions at most recent imaging (Fig. 2). Plain film radiography showed impressive sclerosis of her cysts, with no increase in size (Fig. 2C). Hip pain that was present prior to denosumab initiation resolved rapidly over the course of the first month of treatment with denosumab. She continues to be pain‐free in her hip and has had no issues ambulating in the 13 months since discontinuation of denosumab.

**Figure 1:**
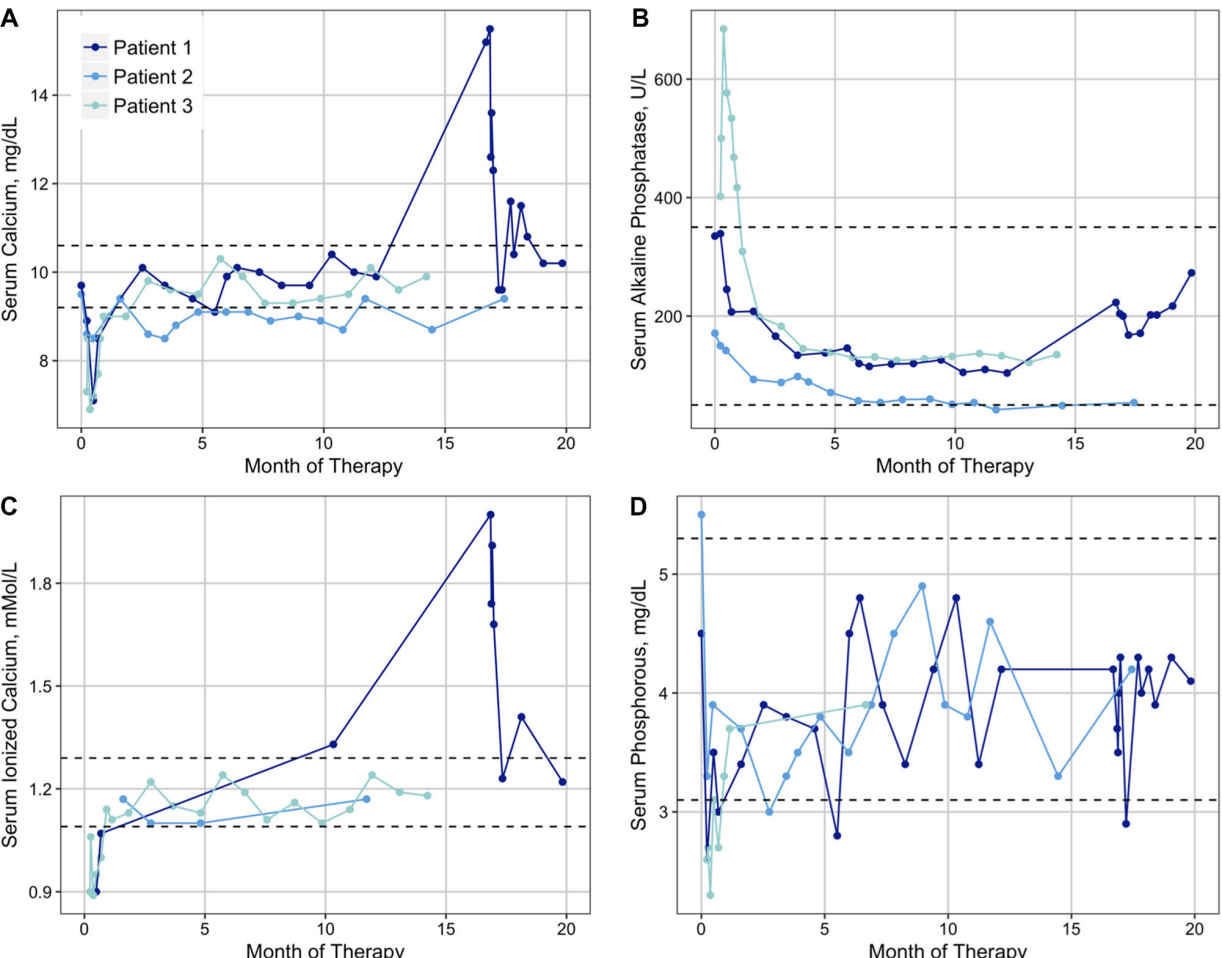
Patient laboratory studies over the duration of denosumab therapy for serum calcium (*A*), alkaline phosphate (*B*), ionized calcium (*C*), and phosphate (*D*). Dashed lines represent laboratory reference ranges at the University of California Los Angeles.

**Figure 2:**
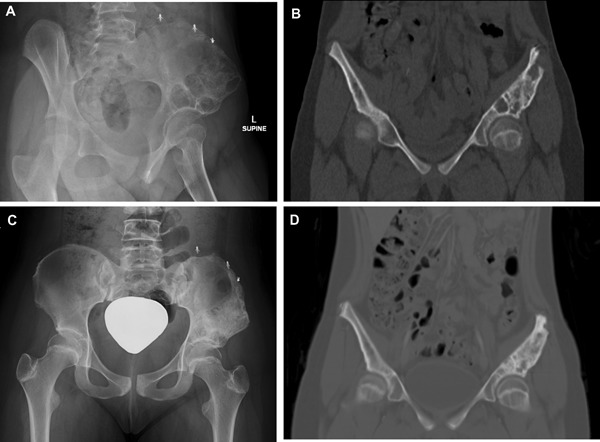
Patient with aneurysmal bone cyst of pelvis. (*A*, *B*) Judet plain film and sagittal CT before denosumab therapy and after two prior surgical interventions (*C*, *D*) and posteroanterior pelvis plain film and sagittal CT after denosumab therapy.

Toxicities associated with treatment included Common Terminology Criteria for Adverse Events (CTCAE v4.0) grade 2 hypocalcemia at the start of therapy and rebound grade 3 to 4 hypercalcemia upon cessation of denosumab, which are described here. In the first month of therapy, the patient experienced asymptomatic hypocalcemia (Fig. 1 *A, C*) with associated hypophosphatemia (Fig. 1 *D*). Her serum calcium normalized within 2 months without any intervention. Five months after her final dose, she was admitted with serum calcium of 15.5 mg/dL (Fig. 1 *A, C*) and symptoms of hypercalcemia, including severe diffuse abdominal pain, nausea, and vomiting. She had no laboratory or radiological evidence of nephrocalcinosis. She required bisphosphonates, furosemide, and calcitonin to lower her serum calcium levels. After discharge, her serum calcium had returned to normal at 9.6. She experienced two episodes of asymptomatic hypercalcemia the following month to 11.6 and 11.5mg/dL, respectively, that corrected with 2 weeks of furosemide therapy. She has subsequently had normal serum calcium levels.

### Patient 2: central giant cell granuloma of the mandible

This patient presented with a progressive mandibular mass over the course of 4 years that was diagnosed as CGCG at 14 years of age. He experienced a recurrence of CGCG with free‐floating lower teeth and severe pain with mastication. Given the progressive severe pain, failure of intralesional steroids and calcitonin, and significant functional limitation and progressive growth of the mass, he was considered for a partial mandibulectomy. Because of the potential morbidity from this surgical procedure, denosumab was discussed as a potential way to avoid or delay the need for mandibulectomy. He initiated denosumab therapy when he was 14 years old, and received monthly denosumab with loading doses at day 8 and day 15 for a total of 15 doses of 120 mg over the course of a year. He weighed 56 kg at the start of therapy. He did not experience any complications while on denosumab or after the completion of therapy, and his laboratory studies did not demonstrate any marked abnormalities in serum calcium levels (Fig. 1A), and serum alkaline phosphatase was appropriately suppressed to below the normal range while on therapy (Fig. 1 *B*).

**Figure 3:**
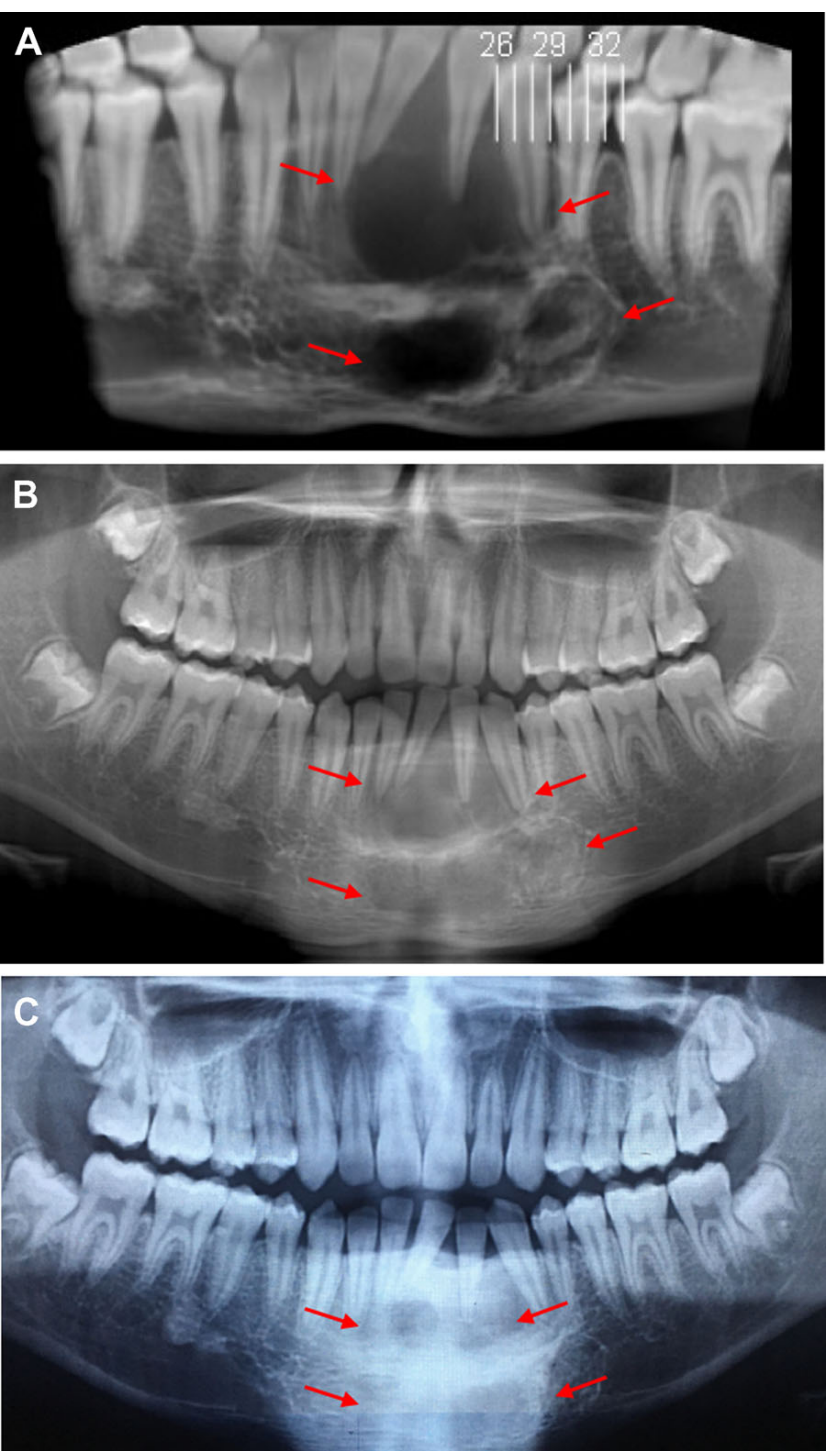
Patient with central giant cell granuloma of the mandible (*A*) before denosumab, (*B*) after 6 months of therapy, and (*C*) after 10 months of therapy.

He had an excellent clinical and radiological response (Fig. 3). Within 1 month of starting denosumab, he had complete resolution of severe mandibular pain; achieved fully functional mastication; and dentition, which was loose and painful, became fixed in the mandible. As with patient 1, serum alkaline phosphatase decreased appropriately with therapy.

### Patient 3: cherubism

This patient was diagnosed with cherubism at 5 years of age, and she experienced refractory progressive disease. She presented with severe pain in her bilateral mandibles with uncontrolled oral–gingival bleeding, resulting in numerous emergency room visits, opioid use, and oral procoagulants (aminocaproic acid). She started denosumab therapy as a 12 year old. She weighed 42 kg at the start of therapy.

**Figure 4:**
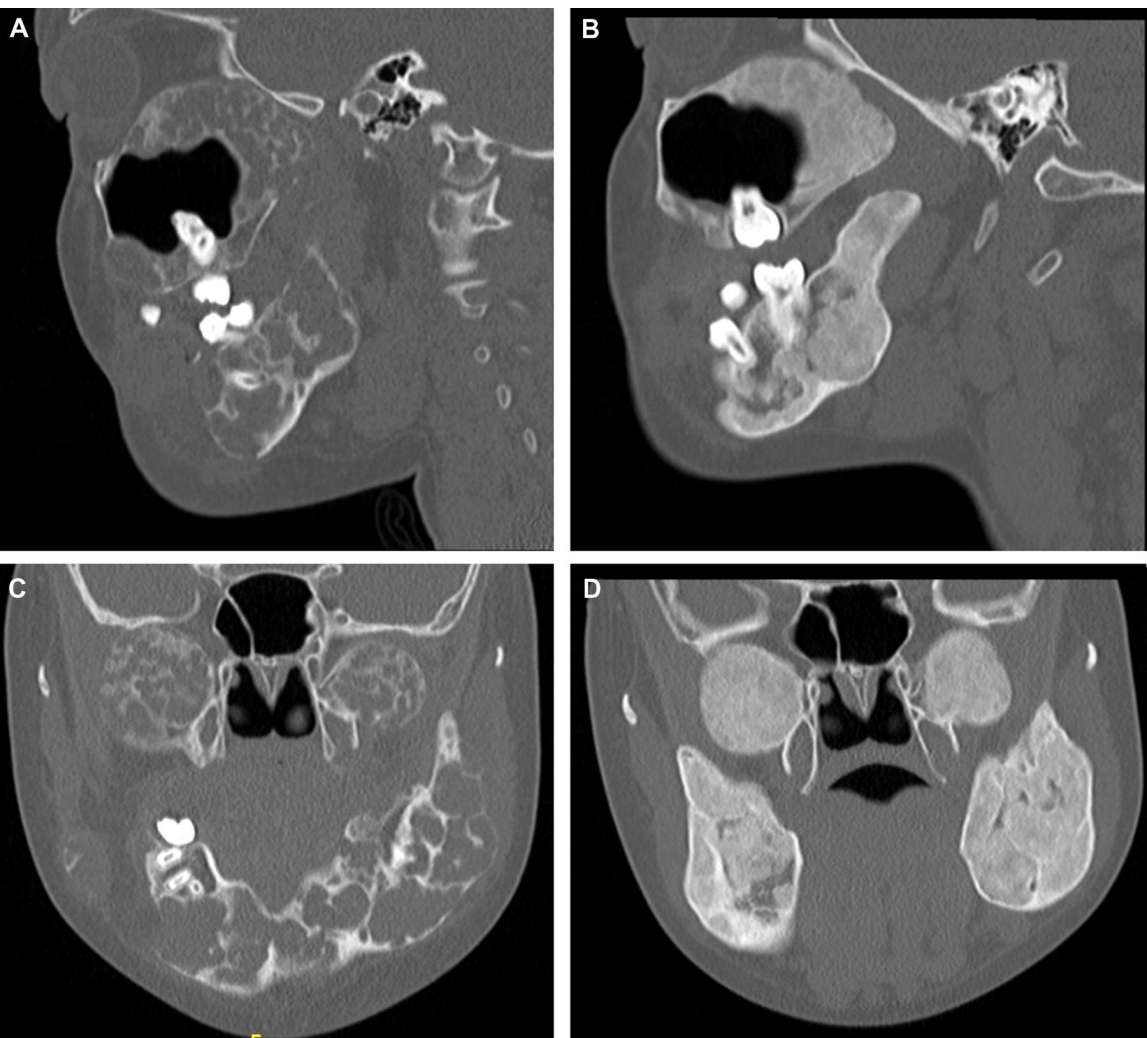
Patient with cherubism (*A*, *C*). Representative sagittal and coronal images before initiation of denosumab (*B*, *D*) and after 6 months of therapy.

Within 1 month of starting denosumab, her pain had completely resolved as did her oral bleeding. After 6 months of therapy, she showed an excellent radiographic response with increased sclerosis in the severely osteolytic mandibular and maxillary lesions (Fig. 4).

Toxicity associated with treatment included serious adverse event CTCAE grade 3 hypocalcemia requiring hospitalization (Fig. 1 *A, C*); symptoms included tingling and numbness in her hands, as well as generalized weakness. She had concomitant hypophosphatemia to 2.6 mg/dL (Fig. 1 *D*) with a normal serum PTH of 48 pg/mL (range 11 to 51 pg/mL). In contrast to patients 1 and 2, serum alkaline phosphatase initially increased in response to denosumab therapy before subsequently decreasing (Fig. 1 *B*).

After this episode, denosumab was dose‐reduced by 50% to 60 mg monthly. After 10 months of therapy, she was started on a weaning schedule to gradually discontinue denosumab. She received her 11th dose 2 months after her 10th, and her 12th dose 2 months after that. Her 13th dose is planned for 3 months after her 12th; her final dose will be 4 months after the previous dose.

## Discussion

In this case series, we describe the off‐label use of denosumab in 3 pediatric patients with recurrent ABC, CGCG, and cherubism. All patients demonstrated a robust and rapid clinical and radiographic response to denosumab. However, 2 patients experienced hypocalcemia following initiation of therapy, with 1 patient progressing to symptomatic hypercalcemia requiring inpatient hospitalization, as well as i.v. bisphosphonate and calcitonin therapy to normalize serum calcium levels. This is similar to a pediatric case that has been described previously.^21^ Our proposed method of avoiding hypercalcemia in pediatric patients is by gradually increasing the time between doses before completely terminating therapy.

Previous studies have used different dosing schedules for pediatric patients. Examples include a starting dose of 1.2 mg/kg, increasing by 0.1 kg/mg weekly, to a target dose of 1.6 mg/kg given monthly after the loading dose.^12^ Others have used a starting dose of 1 mg/kg, increasing by 0.25 mg/kg every 3 months to a final dose 1.75 mg/kg.^21^ Others have opted for a surface area‐based adjustment of 70 mg/m^2^ in children.^7^ One 9‐year‐old pediatric patient was treated with a full adult dose of 120 mg for 18 months.^17^ Recently at our institution, we have eliminated loading doses of denosumab to avoid hypocalcemia with the initiation of therapy.

In the 3 patients described here, hypocalcemia and hypercalcemia were the primary complications noted. In the 22 other published cases of off‐label denosumab use in pediatric and adult patients (Table 1),^7^, ^9^, ^10^, ^11^, ^12^, ^13^, ^14^, ^15^, ^16^, ^17^, ^18^, ^19^, ^20^, ^21^ complications described include symptomatic and asymptomatic hypercalcemia (2 patients, 1 with FD, 1 with CGCG), asymptomatic hypocalcemia (3 patients, 2 with FD, 1 with ABC), asymptomatic hypophosphatemia (3 patients with FD), extremity/skeletal pain (1 patient with CGCG), and delayed wound healing (1 patient with CGCG). In the only other case of symptomatic hypercalcemia described previously, also in a pediatric patient, the hypercalcemia was preceded by hypophosphatemia and increased serum PTH upon initiation of therapy. Our patient who experienced rebound hypercalcemia initially experienced asymptomatic hypocalcemia and hypophosphatemia upon initiation of therapy.

**Table 1:** Summary of complications from previous studies regarding the use of Denosumab in ABC, CGCG, Cherubism and FD

	Study	Year	# Patientsts	Age (years)	Tumor location	Complications
Aneurysmal bone cyst						
Lange et al.^7^		2013	2	8 and 11	Cervical spine	Asymptomatic hypocalcemia in 8‐year‐old
Pauli et al.^13^		2014	1	21	Forearm	None
Pelle et al.^12^		2014	1	5	Sacrum	None
Skubitz et al.^11^		2015	1	27	Sacrum	None
Dubory et al.^10^		2016	1	26	Cervical spine	None
Ghermandi et al.^9^		2016	2	16 and 42	Lumbar spine	None
Central giant cell granuloma						
Schreuder et al.^18^		2014	1	25	Maxilla	Fatigue, myalgia, dizziness, constipation, and cramps
Naidu et al.^17^		2014	2	9 and 42	Mandible	Asymptomatic hypercalcemia in 9‐year‐old
Gupta et al.^16^		2015	1	33	Mandible	None
O'Connell et al.^15^		2015	1	18	Mandible	Shoulder, sternal and neck pain
Bredell et al.^14^		2018	5	4, 18, 19, 22, 26	Mandible (3 patients), maxilla (1), mandible, and maxilla (1)	Delayed wound healing following surgery while on drug in 18‐year‐old
Cherubism						
Eller‐Vainicher et al.^19^		2016	1	20	Mandible	None
Fibrous dysplasia						
Boyce et al.^21^		2012	1	9	Femur	Asymptomatic hypophosphatemia with initiation, severe hypercalcemia (vomiting) on discontinuation, required inpatient admission
Ganda and Seibel^20^		2013	2	44 and 48	Polyostotic	Asymptomatic hypocalcemia and hypophosphatemia

Rebound hypercalcemia may be related to elevated PTH and PTH receptors in people with recently closed growth plates or certain pathologies. Unfortunately, we cannot correlate this theory with the cases described here as serum PTH levels were not routinely measured. In addition, hypophosphatemia and/or hypocalcemia upon initiation of therapy may signal potential problems with rebound hypercalcemia upon cessation of therapy. Moreover, in the patient with cherubism, hypocalcemia was associated with an inappropriately normal serum PTH concentration. This particular patient also displayed higher serum alkaline phosphatase levels at baseline and an initial increase—rather than the typically observed decrease—in alkaline phosphatase activity in response to denosumab. Although further studies are warranted to determine whether these findings are consistent in patients with SH3BP2 mutations, previous reports suggest that cherubism may be associated with impaired osteoblast function and altered PTHrP‐PTHrP receptor interactions,^23^, ^24^ suggesting that an underlying disease mutation may contribute to accentuated changes in mineral ion homeostasis in some patients.

## Conclusions

In the cases described here, denosumab therapy results in significant clinical and radiographic improvement for pediatric patients with ABC, CGCG, and cherubism. In younger patients, problems with serum calcium levels both at the initiation of treatment and at discontinuation may be more common, with symptomatic and protracted rebound hypercalcemia after cessation of therapy the most significant. We present a potential solution to this problem involving the progressive lengthening of time between doses from 1 to 4 months in 1‐month increments before final cessation of denosumab. We also recommend potentially reducing or eliminating loading doses to avoid hypocalcemia with the initiation of therapy. Denosumab may be a viable therapeutic option for children with nonresectable osteoclastic bone dysplasias such as ABC, CGCG, cherubism, and FD. However, because of potential serious adverse events from alterations in calcium homeostasis, this option should be explored carefully in prospective clinical trials of denosumab in children with the aforementioned diseases. The incidence of treatment‐related and treatment‐emergent adverse events with rebound hypercalcemia and hypocalcemia in our patients warrants careful oversight and frequent serial monitoring when administering denosumab and discontinuing denosumab in children and adolescents.

## Disclosures

None of the authors has an actual or perceived conflict of interest with this work.
